# Causes and Cures of Crime

**Published:** 1914-11

**Authors:** Malone Duggan


### Book Reviews.

Causes and Cures of Crime. By Thomas Speed Mosby, Member of the American Bar; Former Pardon Attorney of the State of Missouri; Member American Institute of Criminal Law and Criminology; Author of "Capital Punishment," "Youthful Criminals," "Alcoholism and Crime," "Mothers of Bad Boys," and other essays. C. V. Mosby Company, St. Louis, Publishers. Price, $2.00.

This little volume is a book for the times. While not a pioneer in this branch of learning, it fully and clearly represents the mature judgment of the extensive research recently made along these lines. The author states in his preface that the suppression of crime is a problem for physicians and economists. "That the habits of life and thought which make for health of mind and body likewise militate against crime; that physical, social and moral diseases abide in the same diatheses; that there is a physical basis of morals, as well as a spiritual basis of life; and that, in the study of normal and abnormal man, we can no more consider him as an animal without a soul than as a purely mental phenomenon without a physiological basis." On this high conception the whole subject is considered. He discusses the cosmic, social and individual factor of crime, together with its prevention and cure. Eugenics is rationally considered, and the chapter on education should be generally used by all teachers and parents in the instruction of pupils. Equality, justice, morals and responsibility of the State are admirably presented. He portrays the advanced ideas in reformation and the new penology. Retaliation, intimidations and expiration as a basis of punishment, the author says, is no longer tenable, and in its place he would substitute individualization of punishment, based on a psychological classification; mental and physicial training with a graduated scale of rewards and punishment. The penitentiary, he says, must give way to the reformatory.

In fact, this is one book that should not only be read but studied by every physician and educator, and its principles thoroughly disseminated for the important general enlightenment it furnishes.

MALONE DUGGAN, M. D.

